# Spatio-Temporal Dependence of the Signaling Response in Immune-Receptor Trafficking Networks Regulated by Cell Density: A Theoretical Model

**DOI:** 10.1371/journal.pone.0021786

**Published:** 2011-07-14

**Authors:** Pilar García-Peñarrubia, Juan J. Gálvez, Jesús Gálvez

**Affiliations:** 1 Department of Biochemistry and Molecular Biology and Immunology, School of Medicine, University of Murcia, Murcia, Spain; 2 Department of Information and Communications Engineering, Computer Science Faculty, University of Murcia, Murcia, Spain; 3 Department of Physical Chemistry, Faculty of Chemistry, University of Murcia, Murcia, Spain; Center for Genomic Regulation, Spain

## Abstract

Cell signaling processes involve receptor trafficking through highly connected networks of interacting components. The binding of surface receptors to their specific ligands is a key factor for the control and triggering of signaling pathways. In most experimental systems, ligand concentration and cell density vary within a wide range of values. Dependence of the signal response on cell density is related with the extracellular volume available per cell. This dependence has previously been studied using non-spatial models which assume that signaling components are well mixed and uniformly distributed in a single compartment. In this paper, a mathematical model that shows the influence exerted by cell density on the spatio-temporal evolution of ligands, cell surface receptors, and intracellular signaling molecules is developed. To this end, partial differential equations were used to model ligand and receptor trafficking dynamics through the different domains of the whole system. This enabled us to analyze several interesting features involved with these systems, namely: a) how the perturbation caused by the signaling response propagates through the system; b) receptor internalization dynamics and how cell density affects the robustness of dose-response curves upon variation of the binding affinity; and c) that enhanced correlations between ligand input and system response are obtained under conditions that result in larger perturbations of the equilibrium 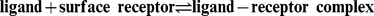. Finally, the results are compared with those obtained by considering that the above components are well mixed in a single compartment.

## Introduction

A characteristic feature of biological systems is their modularity [1]–[4]. Cellular signaling processes, such as those involved in EGFR (Epidermal Growth Factor Receptor), TfR (Transferrin Receptor), LDLR (Low Density Lipoprotein Receptor), and VtgR (Vitellogenin Receptor) systems, exhibit this functionality [5]–[9]. Modules can be considered as subnetworks designed to perform specific functions that often display counter-intuitive behavior, which means that their understanding requires the development of predictive mathematical models. Thus, a particular network can perform different tasks, depending on the values of the set of internal parameters for this module. In addition, the spatial localization (intracellular, superficial, or extracellular) of the molecules that take part in the network must be taken into account [10]–[14]. However, this is difficult and so most theoretical treatments adopt the simplification of considering average concentration values within well-mixed compartments, resulting in ordinary differential equations (ODEs) that can be efficiently solved numerically and require less computing time [15]. Conversely, spatial models start with a geometrical representation of the cell and its environment, and explicitly consider the diffusion of the molecules and their reactions within this geometry. However, this means that the signaling components are not only a function of time, but also of space. Hence, in order to observe the spatio-temporal evolution of these signaling components it is necessary to solve a set of partial differential equations (PDEs), which is much more demanding of computer time than solutions based on ODEs [15], [16]. In fact, non-spatial and spatial models lead to very different mathematical forms, although the initial quantitative mechanistic hypotheses might well be the same [17].

The binding of surface receptors to their specific ligands is a key factor for the control and triggering of signaling pathways [6]–[8], [11], [14], [18]–[20]. As we shall see below, this process can be modeled by designing a module with the appropriate topology and by using the specific set of kinetic parameters for the receptor-ligand system. In most experimental systems, ligand concentration and cell density vary within a wide range of values, so that the signal response dependence on cell density is related with the extracellular volume per cell [6], [21]. Mathematical models of how the signal response is affected by the ligand concentration and cell density have been developed in two recent papers [5], [21]. These have revealed interesting features involved in these processes, including the internalization dynamics of the receptors and how cell density affects the robustness of dose-response curves when the binding affinity varies. However, these models were based on solving a system of coupled ODEs and so, the geometry of the system and their interfaces were not taken into account. The present paper develops a model that permits to study the spatio-temporal dependence of the signaling response in ligand-receptor trafficking networks regulated by cell density. The results obtained provide further insight into the dynamics of the signaling process and suggest that correlation between ligand input and system response increases under conditions that produce larger perturbations of the equilibrium 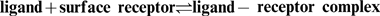. To facilitate comparison with the results obtained from non-spatial models, the starting mechanistic hypotheses are the same as those reported for the general receptor trafficking network developed by Zi and Klipp (M1 in reference (ref.) [21]), and in the design study for signal transduction and ligand transport presented by Shankaran et al.[5].

## Materials and Methods

### Description of the model

**Figure 1:**
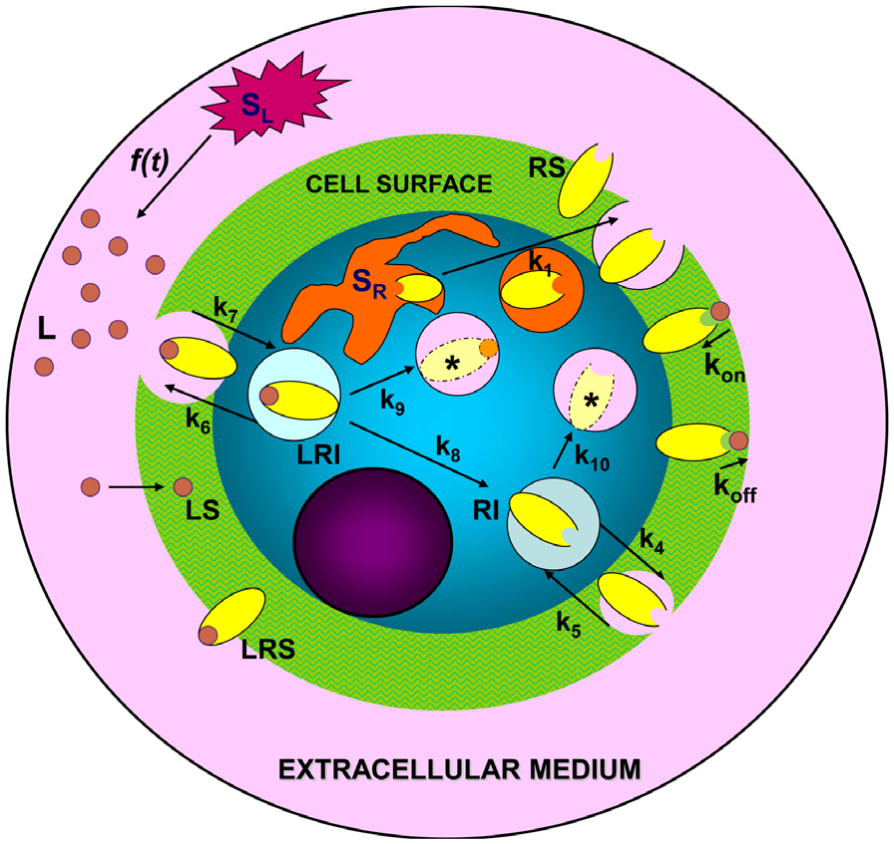
Representation of the model. Schematic representation of the production of ligand and receptors, receptor-ligand binding, internalization, recycling and degradation processes. Regions domains and components are, 1) extracellular medium: L = free ligand; 2) cell surface: LS = free ligand, RS = free receptor, and LRS = receptor-ligand complex; 3) within the cell: RI = internalized receptor, LRI = internalized complex, (*) = degraded products. Ligand is produced at rate 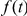 by a source 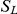, and free receptors are synthesized by the cell at rate 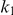. The domains are not drawn to scale especially the cell surface.

Fig.1 shows a scheme of the model and its different spatial domains. There are three different regions separated by their corresponding interfaces: the extracellular volume, the cell surface and the intracellular space. Because this system is non-homogeneous the spatial and temporal evolution of its components have to be considered, which means that a solution of a coupled system of PDEs must be obtained. As mentioned, the characteristics of the solution derived for this trafficking network by considering non-spatial models have been analyzed previously by Zi and Klipp [21] and Shankaran et al [5]. Table 1 shows the nomenclature and units of the kinetic parameters used in these references, as well as the corresponding parameters and units that result in the spatial model shown in this paper. From Fig.1 and Table 1 it follows that 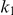 and 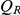 represent the production rate of surface receptors, while 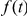 is the corresponding production rate of extracellular ligand. In turn, 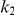 (or 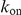) and 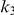 (or 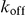) are the association and dissociation rate constants involved in the reversible formation of a receptor-ligand complex at the cell surface, which is formulated according to the mass action law. The rate constants for the internalization of empty and occupied receptors are 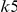 (or 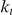) and 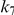 (or 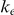), respectively. If these two kinds of receptors are recycled back to the cell surface their rate constants are 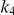 and 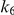 (recycling was not considered in the model of ref.[5]). Finally, within the cell, dephosphorylation of the activated receptors and the degradation of empty and occupied receptors are also considered with rate constants 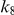, 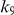 and 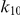 (these processes were omitted in ref.[5]). Inspection of Table 1 reveals that 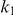 and the four constants involved in internalization and recycling processes, i.e. 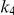 to 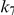, have different units than the corresponding constants used in refs.[5], [21]. This indicates that, unlike the other parameters, 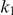 and 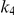 to 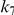 are not equivalent to those that appear in non-spatial models, suggesting that the processes of free receptor synthesis, internalization and recycling must be formulated in different ways in spatial and non-spatial models. From another point of view, these processes are heterogeneous and take place through the interface that separates two different spatial domains (the cell surface and the intracellular space) so that they do not correspond to the homogenous processes that occur in well-mixed compartments. In fact, internalization and recycling are formulated in our model as boundary conditions (see below), while in a well-mixed compartment model they are considered simply as reactions that result in extra terms in the corresponding rate equations. Finally, this formulation, as in ref.[21], also includes particular networks that are obtained by writing some of the rate constants as 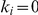 (for instance, recycling processes do not occur when 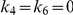).

**Table 1:** Parameter for receptor trafficking networks.

Zi et al 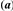	Shankaran et al 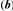	units	this work	units
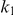	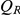	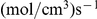	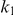	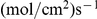
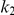	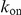	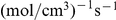	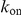	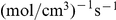
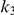	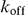	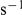	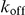	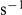
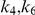	–	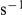	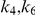	m/s
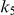	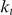	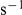	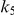	m/s
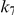	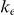	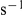	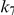	m/s
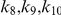	–	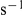	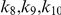	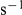
–	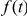	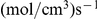	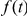	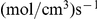

-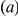 kinetic parameters in the Zi and Klipp model [21]; 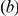 idem in Shankaran, Resat and Wiley's model [5].

-units are expressed as a function of 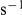, 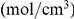, 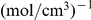 and m/s although other equivalent units (for instance 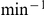, or nM) were also used in the three models.

-processes that were not considered in a given model are shown as (–).

### Model geometry

The domains shown in Fig.1 consist of three spherical regions of radii 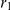, 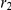, and 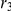 that include the extracellular volume per cell, the cell surface and the cell volume. Extracellular volume is defined inside the region 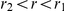, while cell surface and cell volume are confined inside the regions 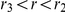 and 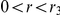, respectively. The value of 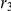 used for computations was that of a typical mammalian cell, i.e. 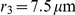 [21]. In turn, for simplicity, the cell surface was modeled as the space between two concentric spherical surfaces separated by 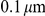, i.e. 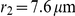. Moreover, the wide experimental range of values of cell density was taken into account by varying the radius of the extracellular domain accordingly (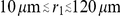). Finally, and because the geometry is defined from the start by considering the values of 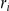 (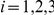) as parameters, a geometric parameter study using different 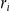-values can easily be performed by re-running the geometry sequence (see below).

### Notation/definitions and formulation of the boundary value problem


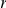
 : distance from the center of the cell


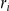
 : external radius of the domain 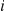 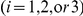



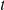
 : time


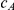
 : concentration of species 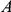 (ligands, receptors, or complexes)


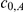
 : initial concentration of species 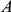



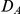
 : diffusion coefficient species 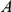



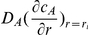
 : flux of species 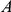 through the interfase 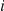 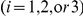


With this notation, and by assuming that spherical diffusion only depends on the radial coordinate, the system of PDEs for the concentration distribution of ligands, receptors and complexes and their initial and boundary conditions in the different domains involved in the model displayed in Fig.1 can be formulated as follows:

### 1) Extracellular volume



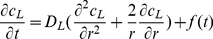
(1)

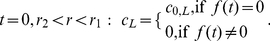
(2)




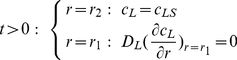
(3)In eqns.(1) and (2), 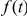 is the ligand input rate, which is zero when a single instantaneous dose of ligand is added to the system, so that at 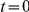 the total concentration is 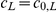. Conversely, 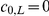 in those cases where 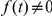. In turn, eqns.(3) show, on the one hand, that at the interface between the extracellular medium and the cell surface, the concentrations of species 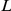 and 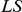 are coupled and, on the other, that there is no outward flux of ligand from the extracellular medium (insulation condition at 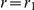).

### 2) Cell surface



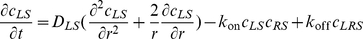
(4)

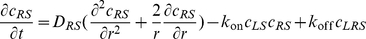
(5)




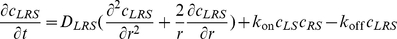
(6)




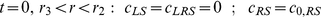
(7)




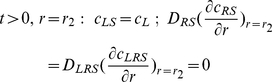
(8)




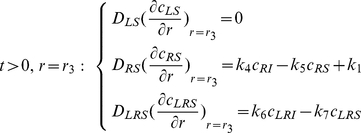
(9)


### 3) Within the cell



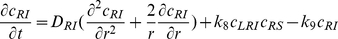
(10)

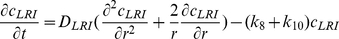
(11)




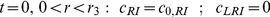
(12)




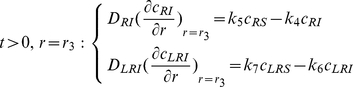
(13)


### Computation of the boundary value problem

The set of PDEs and boundary conditions defined by eqns.(1) – (13) has no analytical solution and must be solved numerically. To this end, the program Comsol 4.0a, which is based on the finite element method, was used. Compared with previous versions, Comsol 4.0a has the advantage that it applies the concept of sequences, i.e. all the steps to create the geometry, mesh, studies and solver settings, as well as the visualization and presentation of results, are recorded when the model is built. It is therefore easy to parameterize any part of the model by changing a node in the model tree and rerunning the sequences. Also, convergence tests for the computed results were performed by refining the mesh and running the study again to assess whether the solution converges to a stable value as the mesh is refined.

### Simulation assays: Values of kinetic parameters, ligand concentration and cell density

Because of simplifications in the model from ref.[5], our spatial model was mainly compared with the model from ref.[21]. The parameters and initial conditions used for computation in most simulations are listed in Table 2, so that when dependence on a given parameter was studied, the other parameter values were kept constant. As regards the values of the diffusion coefficients which are necessary for computation (see eqns.(1) – (13)), typical values were taken from the literature [22]–[24]: at the cell surface, 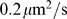 for species not forming complexes, and 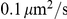 for complexes. Within the cell these values were 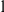 and 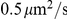, respectively. For ligand in the extracellular medium, a value of 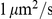 was considered. Finally, cell densities and ligand concentration were varied in a wide range to cover most experimental conditions.

**Table 2:** Kinetic parameters for the different processes displayed in Figure 1.

rate constants values used for computation			
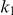	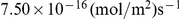	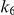	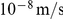
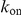	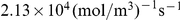	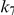	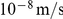
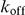	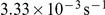	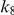	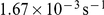
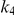	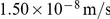	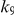	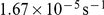
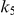	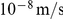	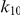	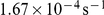

-Units are given in the SI system although most results were expressed in conventional units, for instance, nM instead of 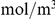.

-The rate constants 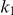 and 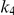 to 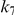 are related to processes that take place through the interface that separates the cell surface and the inside the cell and do not correspond with the rate constants used in refs. [21] and [5]. Their values were assigned so that under conditions where spatial gradients of concentration become less significant the solutions computed behave like those obtained by using non-spatial models. For the rest of the trafficking parameters the average values for the EGF signaling pathway reported in ref.[21] were used.

-Initial conditions: 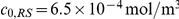, 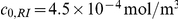. In simulations with 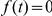 a single input dose of ligand 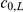 was added to the extracellular medium.

## Results and Discussion

As regards the formulation of the boundary value problem presented in previous subsections, two remarks can be made about eqns.(1) – (13): 1) both internalization and recycling are included in the kinetic equations in well mixed models (eqns.(1) – (4) in ref.[21] and eqns.(1a) in ref.[5]). In our model, however, because internalization and recycling occur through the interface that separates two different domains, these processes are heterogenous and are therefore formulated as boundary conditions (eqns.(9) and (13)). Also, note that the units of the constants (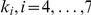) involved in these equations are m/s and not 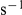 as in non-spatial models. In fact, these four constants are mass transfer coefficients rather than rate constants, revealing that the kinetic behavior of internalization and recycling depends not only on the species involved but also on the properties of the interface (membrane) through which these transport processes occur; 2) in a model of well mixed components the extracellular volume per cell is included in the kinetic equation for the concentration of ligand (see eqn.(5) in ref.[21] and eqn.(1a) in ref.[5]). Conversely, in our spatial model this volume is not involved in the kinetic equations but is modeled in the geometry by changing the value of 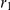. These observations demonstrate clearly that identical mechanistic hypotheses can be formulated in very different ways, depending on the model adopted to describe the system.

### Geometry and cell density

**Figure 2:**
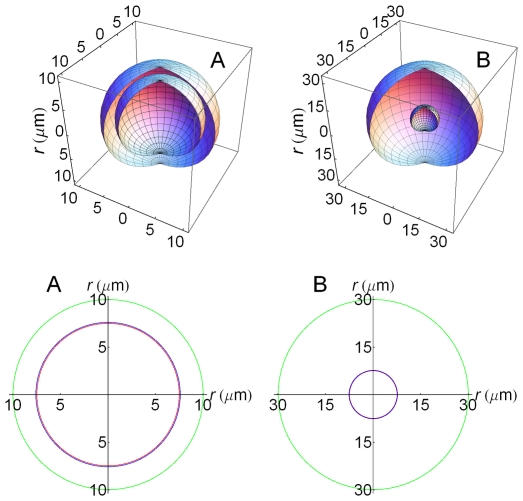
Spatial domains. Top: three-dimensional spatial domains: A) 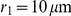, 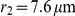, 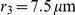. The spherical surfaces 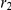 and 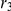 are so close that they appear overlapped; B) 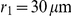, other conditions as in A. Note the different scales in A) and B) although the volume of the cell is the same in both cases. Bottom: A) cross section representation of top domain A. The two circles 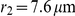 (blue) and 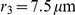 (red) can be now distinguished; B) cross section representation of top domain B.

Since our spatial model has been built with the 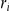-values (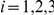) as parameters, geometries are easily modeled and are related with the corresponding cell density values by 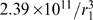 cells/ml when the external radius 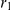 is expressed in 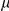
m. Fig.2 illustrates the geometries and spatial domains corresponding to two cases with different cell densities. Thus, Fig.2A in the top panel is a three-dimensional (3D) representation of the cell and the extracellular medium when the cell density is high (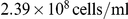). Conversely, in Fig.2B the volume of the extracellular medium is much larger and so the cell density-value is smaller (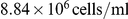). In turn, the corresponding two-dimensional (2D) cross section representations (bottom panel, Figs.2A and 2B) allow a rapid inspection of the relative size of the different domains and the distribution of species in the system (see below).

### Spatio-temporal variation of ligand

**Figure 3:**
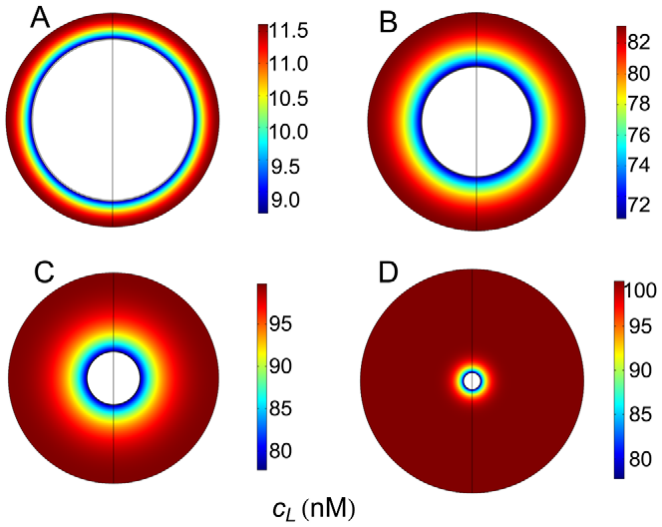
Two-dimensional spatial distributions of ligand at four cell densities. Spatial distribution of ligand computed at 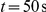, 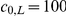 nM, 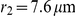, 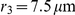, for four cell densities: A) high cell density, 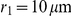; B) medium-high cell density, 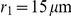; C) medium-low cell density, 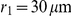; D) low cell density, 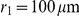. The rate constants used are given in Table 2.

**Figure 4:**
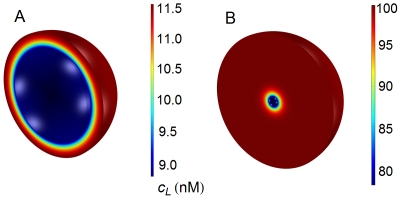
Three-dimensional spatial distributions of ligand at high and low cell densities. Spatial distribution of ligand computed at high (A, 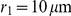) and low (B, 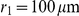) cell densities. Note the difference of scale in both figures although the cell volume does not change. Other conditions as in Fig.3.

The influence exerted by cell density on the spatial distribution of ligand is illustrated in Fig.3, in which 2D plots have been computed at 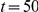 s for four different cell densities ranging from high (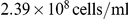) to low (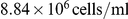) after the addition of a single ligand dose of 100 nM at 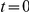. The corresponding 3D plots for high and low cell densities are shown in Fig.4. These figures show that: a) concentration gradients are clearly established and, as expected, ligand is transported by diffusion toward the cell surface; b) since variations in ligand concentration are related to the formation of ligand-receptor complexes, which, in turn, regulate the trafficking response, it follows that cell density must be involved in the regulation of this response (see below); c) when the cell density is high there is a dramatic drop in the concentration of ligand throughout the domain (from 100 nM to 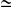
10 nM, panels A in Figs.3 and 4); and d) at low cell densities this drop is smaller, especially in the outer regions of the extracellular medium (panel D in Fig.3 and panel B in Fig.4).

**Figure 5:**
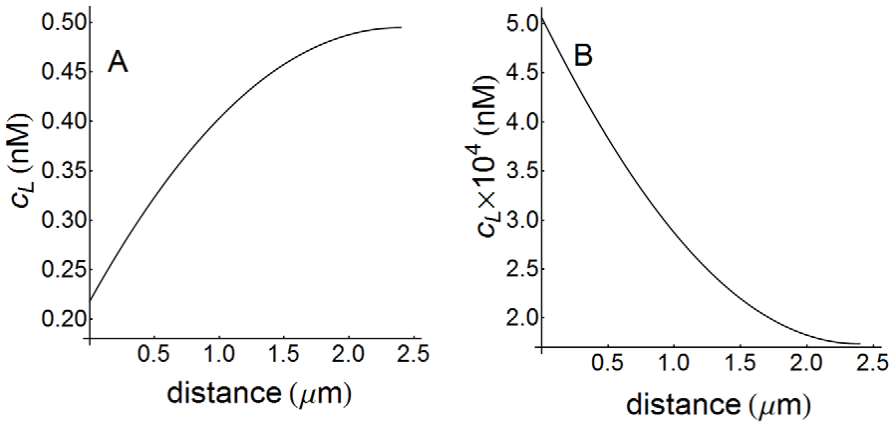
Concentration-distance profiles of ligand at high cell density. Ligand profiles computed as a function of distance at high cell density (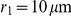) and 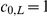 nM for 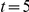 s (A) and 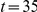 s (B). These profiles are defined in the extracellular medium (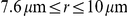) and distance is measured in the radial direction from the cell surface, i.e. 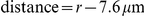. The 2D plots (not shown) viewed from the cell surface appear blue-red in (A) and red-blue in (B).

**Figure 6:**
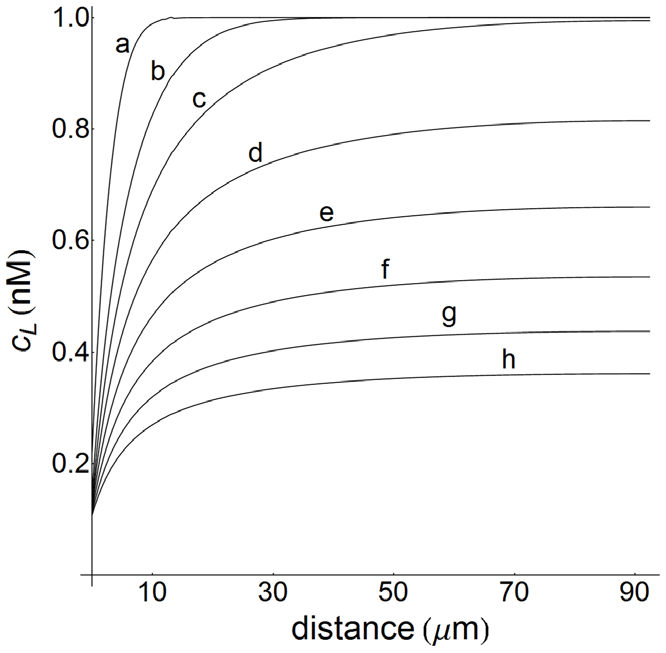
Concentration-distance profiles of ligand at low cell density. Ligand profiles computed as a function of distance at low cell density (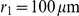) for 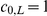 nM. The 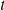-values (s) are: a) 10; b) 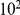; c) 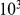; d) 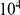; e) 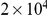; f) 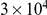; g) 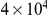; h) 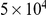. These profiles are defined in the extracellular medium (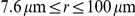) and distance is defined as in Fig.5. The corresponding 2D plots (not shown) viewed from the cell surface appear blue-red.

However, if the initial input of ligand decreases, some unexpected results in the spatio-temporal distribution of ligand are observed at high cell densities. This is illustrated in Fig.5 where concentration-distance profiles of ligand at two different times have been computed for a value of 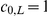 nM. Panels A and B in this figure show that the profiles are well established throughout the extracellular medium, but that the ligand is transported in opposite directions. Thus, while in panel A the profile at 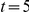 s is as expected, i.e the drop in 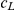-values increases as we approach the cell surface, the corresponding profile in Panel B at 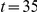 s is inverted. The regular profile at short times indicates that to trigger the signaling process the ligand must reach the cell surface and bind to cell surface receptors, causing a decrease of about 80% of the ligand concentration even at this early stage of the process. At longer times most of the ligand is consumed in the formation of ligand-receptor complexes and its presence in the extracellular medium is only provided by the dissociation of these complexes at the cell surface, which leads to the inversion of the corresponding profile. This is in agreement with the fact that regular profiles always result in the case of low cell densities since, under these conditions, a large supply of ligand is available in the extracellular medium. Fig.6, in which concentration profiles have been computed at low cell density (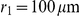) for a wide range of values of 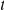, illustrates this situation. These profiles are well developed up to a distance of 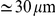 from the cell surface and extend deeper into the extracellular medium as time increases, but no inversion is noticed even at 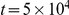 s. Note that the concentration profiles shown in Figs.3, 4, 5, 6 involve ligand distribution as a function of the distance to the cell surface. Therefore, a comparison with profiles obtained from non-spatial models which depend only on time cannot be performed.

**Figure 7:**
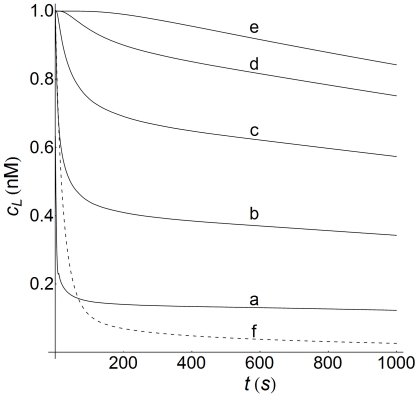
Concentration-time profiles of ligand at medium cell density. Concentration-time profiles of ligand computed at medium cell density (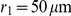) for 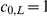 nM. These profiles are defined in the extracellular medium (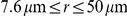) and distance (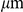) measured from the cell surface in a radial direction is: a) 0; b) 2.4; c) 7.4; d) 17.4; e) 42.4. The dashed line f) is the profile computed with the non-spatial model of ref.[21] using the kinetic parameters given in this reference and in Table 2.

Ligand concentration-time profiles are computed in Fig.7 for a medium value of cell density (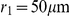) at increasing distances from the cell surface (curves a–e). For comparison, the corresponding plot obtained by using a non-spatial model is also included (dashed curve, f). This figure shows that the behavior of the profiles is similar in both kinds of model, the ligand concentration falling as time increases. Quantitatively, however, the differences are significant except at the cell surface (curve a). Furthermore, it follows that the non-spatial model predicts that the ligand has practically disappeared at 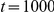 s. Conversely, computations using our spatial model reveal that, at this time, the drop in ligand concentration is only 43% at 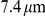 (curve c) and 16% in the outer regions of the extracellular medium (curve e), meaning that a substantial supply of ligand still remains.

### Spatio-temporal variations of receptor and ligand-receptor complexes inside the cell

**Figure 8:**
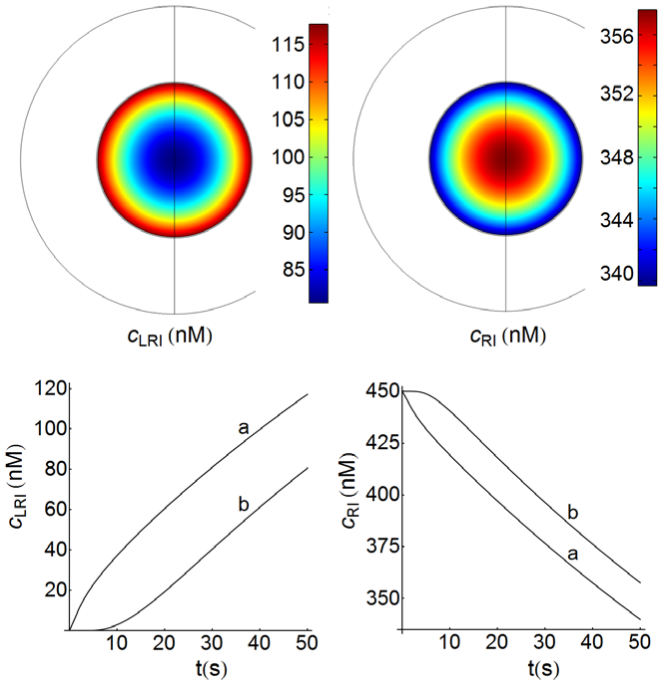
Spatio-temporal distributions of LRI and RI species. Top:2D spatial distributions of internalized ligand-receptor complexes and free receptors (LRI and RI species) computed at medium-high cell density (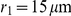) and 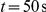. The white space inside the larger circle is the extracellular medium. The concentration gradients of LRI and RI are established in opposite directions. Bottom: concentration-time profiles of LRI and RI at close proximity to the cell surface (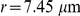, curves a) and at the center of the cell (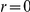, curves b). Other conditions as in Fig.3.

Spatial distributions of internalized ligand-receptor complexes and free receptors (LRI and RI species) computed for a medium-high cell density (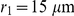) are displayed in Fig.8. Note that under these conditions the flux of free receptors is directed toward the cell surface, while the flux of ligand-receptor complexes is directed toward the inner regions of the cell, i.e. the maximum concentration of LRI species is reached at the interface with the cell surface. This suggests that the decoding of external information transported by these species may also show a spatial dependence. This observation is in accordance with the behavior exhibited by the concentration-time profiles of LRI and RI, which are also displayed in Fig.8. These profiles were computed very close to the cell surface (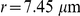, curves a) and in the center of the cell (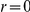, curves b). Note that the LRI profiles show a lag phase at 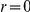, while they exhibit a burst phase close to the cell surface. However, unlike those processes where the presence of lag and/or burst phases is under kinetic control, in this case the appearance of these phases is caused by concentration gradients (spatial control). Similar considerations apply to the RI profiles, although, because the concentration gradients for LRI and RI are established in opposite directions, the greater drop in 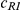-values occurs in the proximity of the cell surface.

### Influence exerted by cell density on the concentrations of free receptor and ligand-receptor complexes at the cell surface and inside the cell

**Figure 9:**
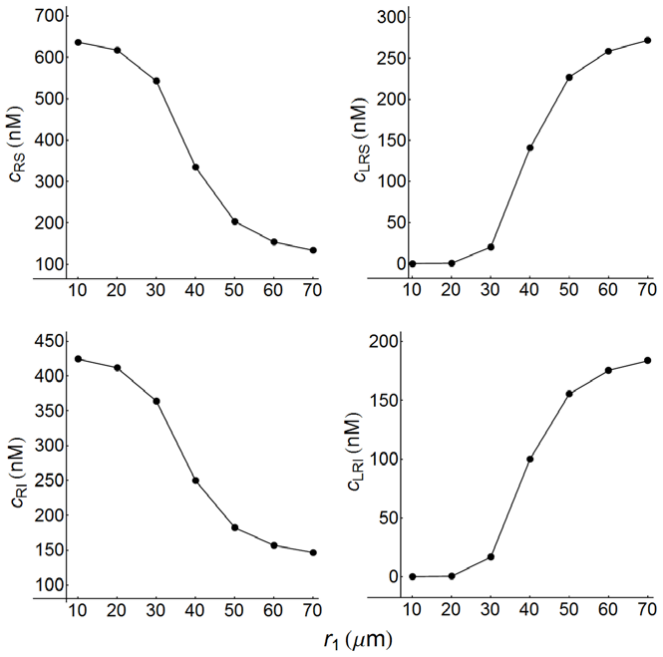
Concentration dependence of surface and internalized receptors and ligand-receptor complexes on cell density. RS, RI, LRS, and LRI concentrations computed as a function of the radius of the extracellular medium per cell, 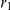. When 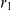 is expressed in 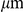 the cell density-values are given by 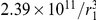 cells/ml. Internalized species (receptors and ligand-receptor complexes) values were computed at the center of the cell (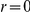), while surface species were determined at 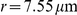. 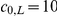 nM, 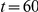 min. Other conditions as in Fig.3.

The concentrations of free receptors and ligand-receptor complexes at the cell surface (RS and LRS species) and inside the cell (RI and LRI species) show a strong dependence on cell density. This is illustrated in Fig.9, in which the concentrations of RS, LRS, RI and LRI have been computed for 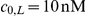 and 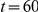 min as a function of the radius of the extracellular medium per cell, 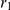. The curves obtained are sigmoid and demonstrate that, among other factors, responses in trafficking networks can be effectively regulated by modifying the cell density.

**Figure 10:**
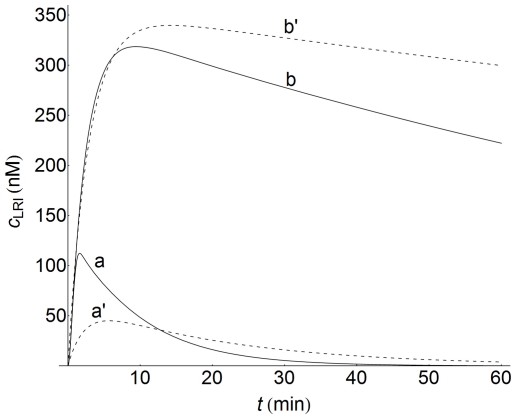
LRI Concentration-time profiles at high and low cell densities. Concentration-time profiles of LRI computed at high (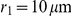, curves a) and low (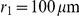, curves b) cell densities. LRI-values were obtained at the center of the cell (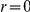). The dashed lines (curves a' and b') are the profiles computed with the non-spatial model of ref.[21] using the kinetic parameters given in this reference and in Table 2. Other conditions as in Fig.3.

Concentration-time profiles of internalized ligand-receptor complexes computed at high and low cell densities confirm this fact, as can be seen in Fig.10 (curves a and b). This figure reveals that a decrease in cell density increases the value of the maximum response, although the time at which this maximum value is attained is delayed compared with that observed at a high cell density. The profiles for surface ligand-receptor complexes behave in the same way (data not shown). For comparison, in Fig.10 the corresponding plots obtained using a non-spatial model are also shown (dashed curves, a' and b'). Although the profiles obtained with both models are similar, there are significant quantitative differences at high cell density values, especially during the early stages of the response (curves a). Conversely, these differences are observed at longer times when the values of cell density decrease (curves b).

**Figure 11:**
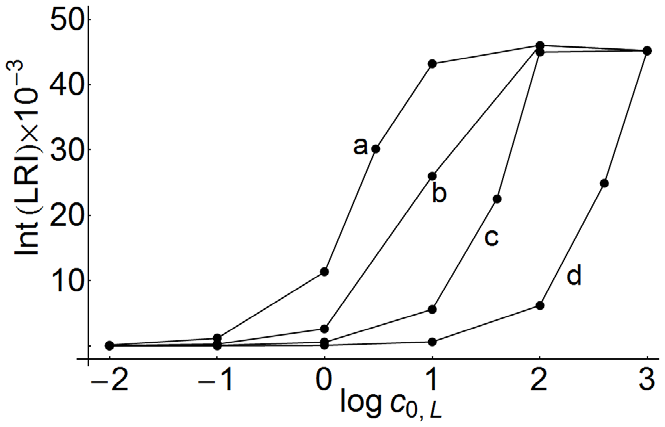
Dose-response curves of LRI computed at different cell densities. The LRI response was computed at the center of the cell (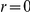) and expressed as the area under the concentration-time profiles for 10 hours. Integration was performed expressing the concentrations of 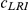 in nM and times in min. The ligand concentration 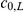 is given in nM. The values of 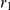 (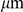) are: a) 100; b) 50; c) 30; d) 15.



On the other hand, it has been suggested [5] that the extracellular volume presents unique characteristics since it is independent on receptor and ligand properties, so that, a given receptor-ligand system presumably evolves to be optimized and performs its functions in a defined range of volumes. For this reason it is interesting to obtain dose-response curves as a function of cell density. The results determined for the set of parameters given in Table 2 are displayed in Fig.11, where they are expressed as integrated responses. These responses were calculated by computing the area under the concentration-time profiles

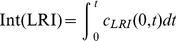
(14)in the center of the cell (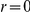) over a period of 10 hours, which is the period of time used in ref.[21] to calculate the same response. The curves obtained are sigmoid and shift toward higher ligand concentrations as the cell density increases. Integrated responses computed for surface ligand-receptor complexes behave similarly (data not shown), indicating that these kinds of curves are useful for determining the robustness and sensitivity of a system to changes in the extracellular volume.

### Internalization and endocytic downregulation

**Figure 12:**
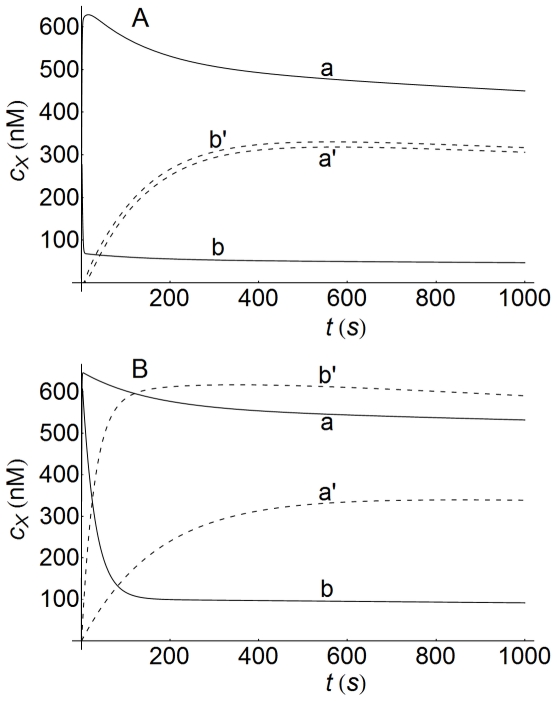
Concentration-time profiles of LRS and LRI with and without endocytic downregulation. Concentration-time profiles of LRS and LRI computed at two values of the endocytic downregulation ratio 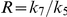: curves (a,a') 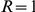, curves (b,b') 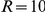. X = LRS (solid lines), X = LRI (dashed lines). Panel A: curves obtained using the spatial model with 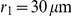. The spatial domain for LRS is 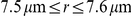 and for LRI 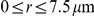. The time profiles were computed at 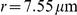 for LRS and at the center of the cell (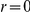) for LRI. The parameters used for computation are those given in Table 2, except curves b and b' which were obtained with a 10-fold increase in the 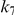-value. Panel B: curves computed using the non-spatial model of ref.[21] and the kinetic parameters given in this reference and in Table 2. Curves (a,a') 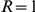, curves (b,b') 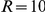.

It has been suggested that the internalization of empty and occupied receptors, as well as occupancy-induced receptor loss (endocytic downregulation), may enhance the function of signaling receptors [5], [21], [25], [26]. In panel A of Fig.12 we have computed the corresponding responses for surface (LRS) and internalized (LRI) complexes for two values of the ratio 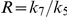, which has been proposed as an estimate for quantifying ligand-induced endocytosis [5]. By comparing the LRS responses in the absence of endocytic downregulation (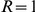), and when induced endocytosis occurs (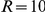), we find that endocytic downregulation exerts a strong influence on the time course of LRS (solid curves). Thus, for 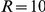, the response is faster although the peak-value and the decay decrease dramatically (Fig.12, panel A, curves a and b). Conversely, the corresponding LRI responses (dashed curves) reveal that the effect exerted by endocytic downregulation is very small. Also, if these results are compared with those obtained with a well-mixed model (Fig.12, panel B) it is seen that the LRS responses behave similarly in both models while, conversely, the LRI response is quite different. Thus, the LRI profiles in panel A exhibit a lag phase due to a spatial control that is not present in the curves of panel B. In addition, panel A shows the low degree of sensitivity of the LRI profiles to changes in the R-ratio, while in panel B, the peak value of LRI at 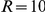 is reached faster and is about 100% greater than the corresponding peak value obtained at 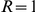. This observation reveals an important fact that must be borne in mind, namely, that in well-mixed models, kinetic equations for concentrations of receptors and ligand-receptor complexes, whether internalized or not, refer to the same volume [5], [21]. Conversely, in spatial models these concentrations refer to the volume of their corresponding domains. Thus, for instance, from Table 2 we see that at 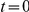 the surface concentration of free receptors is 650 nM (this amounts to 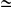
40 molecules/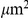 in our cell surface model). By assuming that the available ligand concentration is not limiting for the formation of ligand-receptor complexes, i.e. that all surface complexes can be converted into internalized complexes, the mass balance for the 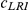 value obtained by using a well mixed model is 650 nM. However, when the volume of the occupied domains are taken into account the corresponding mass balance shows that the maximum value of 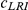 under the same conditions is only 26.3 nM. In addition, if we include the concentration of internalized receptors (450 nM, see Table 2) in these calculations, the corresponding maximum 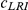 values in both types of models are 1100 nM and 476.3 nM, respectively. In short, receptor trafficking networks are the result of a complex mass balance due to the different volumes of the domains involved. This must be taken into consideration to avoid misleading interpretations when comparing data from different models and experiments.

### Recycling and signaling processes

**Figure 13:**
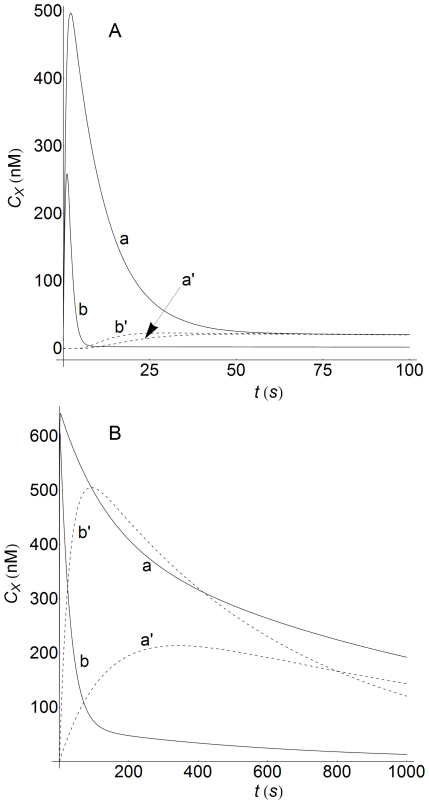
Concentration-time profiles of LRS and LRI without recycling of internalized receptors. Concentration-time profiles of LRS and LRI computed at two values of the endocytic downregulation ratio 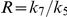: curves (a,a') 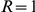, curves (b,b') 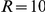. X = LRS (solid lines), X = LRI (dashed lines). There is no recycling of empty receptors to cell surface (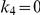). Panel A: curves obtained using the spatial model. Panel B: curves obtained with the non-spatial model of ref.[21]. Other conditions for panels A and B as in Fig.12.

The influence exerted on the signaling process by the recycling of empty and occupied receptors (RI and LRI species) to the cell surface is determined by the values of the constants 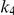 and 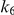. If these constants are not zero, recycling occurs and the response curves obtained are similar to those shown above in Fig.12. The curves computed for the LRS and LRI responses when only the recycling of empty receptors is prevented (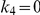) are displayed in Fig.13 for both kinds of model. The curves obtained for 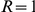 and 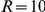 show that the inhibition of recycling causes a faster decay of the LRS and LRI responses, as well as a decrease in their amplitudes (compare panels A in Figs.12 and 13 and note the different time scales in both figures). As in Fig.12, increasing the value of 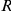 results in greater differences in the LRS responses, although these differences become almost negligible in the case of internalized complexes. As regards the results obtained with the non-spatial model, it follows that the absence of recycling also produces a decrease in the corresponding responses, although these reductions are much smaller than those obtained with the spatial model (see panels B in Figs.12 and 13). As mentioned above, these results can be attributed to the different mass balances involved of both models. Also, note the large differences between the peak values for the LRI responses obtained with and without recycling (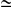 300 nM and 26 nM, respectively, see curves (a' and b') in panels A in Figs.12 and 13). This can be attributed to the fact that, when 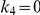, the receptors inside the cell are not able to engage in the formation of surface complexes, which, after internalization, would result in the enhancement of the LRI response. If, in addition to 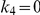, we consider 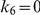, i.e. that the recycling of occupied receptors is also prevented, the results obtained do not differ significantly from those computed when only 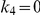. This suggests that the influence on the LRS and LRI responses exerted by the recycling process is mainly determined by the number of empty receptors within the cell at the beginning of the signaling process.

### Concentration gradients

**Figure 14:**
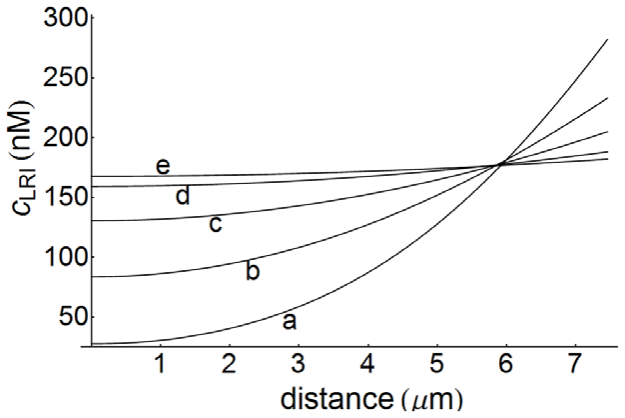
Concentration-distance profiles of LRI as a function of 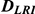. Concentration-distance profiles of internalized ligand-receptor complexes computed for 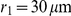 (medium cell density) and 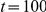 s as a function of diffusion coefficient. These profiles are defined inside the cell (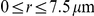) and distance is measured in the radial direction from the center of the cell. The values of the diffusion coefficient 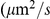) are: a) 0.05; b) 0.1; c) 0.2; d) 0.5; e) 1. Other conditions as in Fig.3.

Concentration gradients are inherent to spatial models, where they are determined by the transport properties of the signaling components in the different domains [13]. To examine this, the influence exerted by the diffusion coefficient of internalized ligand-receptor complexes on the LRI response was studied. The results obtained are displayed in Fig.14, which shows that concentration gradients are established in all cases, and that more pronounced gradients are obtained as transport in the intracellular medium becomes more difficult (lower diffusion coefficients). Also, concentration gradients for ligand have been computed in the extracellular medium by modifying the values of the diffusion coefficient 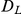 between 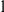 and 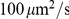. The computed curves show the presence of well defined gradients that, as in the case of the LRI complexes, increase as 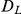 becomes lower (data not shown). Since these gradients influence the response levels, it is clear that a quantitative description of a given system should take into account its transport properties.

### Dynamics of the signaling response

**Figure 15:**
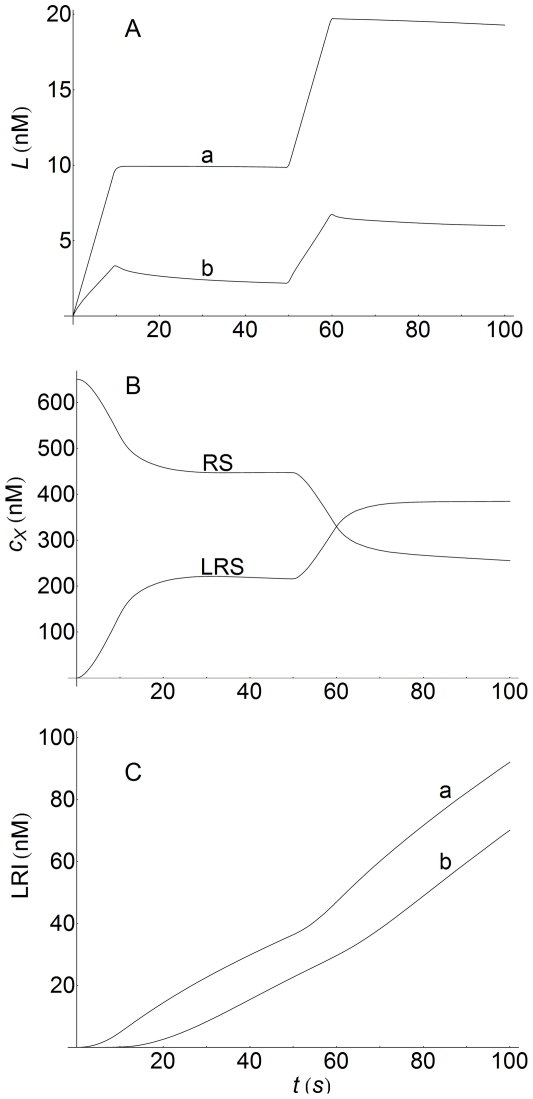
Extracellular, surface, and internalized response to step changes in ligand input rate. Concentration responses of L, RS, LRS, and LRI to step changes in the ligand input rate. Two inputs of 10 s duration separated by a recovery phase of 40 s were considered. In both inputs ligand enters the system at a constant rate of 1 nM/s. 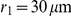, 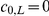, 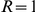. Panel A: ligand response computed at the outer region of the extracellular medium (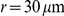, curve a) and at the interface with the cell surface (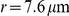, curve b). Panel B: concentration-time profiles of empty (X = RS) and occupied (X = LRS) cell surface receptors obtained at 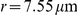. Panel C: concentration-time profiles of internalized ligand-receptor complexes computed at the surface of the cell (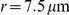, curve a) and in the center of the cell (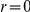, curve b). Other conditions as in Fig.3.

The responses of the system to step inputs of ligand were also tested. In Fig.15 two ligand inputs of 10 s duration, during which ligand enters the system at a constant rate of 1 nM/s, were considered. These inputs were separated by a recovery phase of 40 s when the ligand entry rate was zero. The results obtained are illustrative because they provide a good description of how the perturbation (input ligand) propagates through the system. Thus, in the outer regions of the extracellular medium (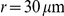, Fig.15, Panel A, curve a), far from the the interface with the cell surface (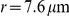, Fig.15, Panel A, curve b), perturbations caused by the presence of this interface do not operate and ligand concentration increases at a rate of 1 nM/s, so that at the end of the first input (10 s), 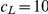 nM. This concentration level is maintained during the recovery phase (40 s), after which a second stimulation phase of ligand input begins. As a result, 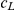 increases again at the same rate of 1 nM/s and, at the end of the second ligand input, 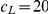 nM. This value of 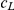 does not change significantly at longer times (see curve a in Panel A, Fig.15). However, this behavior is quite different at the interface with the cell surface. In this region, although the ligand also enters at a rate of 1 nM/s, the slope of the curve obtained is less pronounced and, as a result, at the end of the first stimulation phase the value of 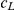 is only 3.32 nM (curve b in Panel A). This is a consequence of the perturbation caused by interactions between ligand and surface receptors to form ligand-receptor complexes, the effect of which is a decrease in the values of 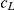. These opposing effects (input and depletion of ligand) produce a net increase of 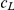 during the stimulation phase, but with a less pronounced slope than when complexes are not formed. During the recovery phase (with a ligand entry rate of zero) surface ligand-receptor complexes continue to be formed and, therefore, the levels of 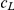 continue to diminish. At 50 s the second ligand input starts and this new perturbation increases the values of 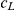 until the end of the input (60 s). These differences in the 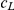-profiles, at the cell surface and at the outer end of the extracellular medium (curves a and b in Panel A), cause concentration gradients that transport ligand from the outer regions toward the interface. This process will continue at varying rates until the ligand and/or surface receptors are depleted, or until the system reaches equilibrium.

In turn, the response of empty and occupied surface to ligand input receptors is shown in Panel B. In this case, the analysis of changes in the values of 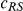 and 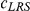 caused by the perturbation of input ligand is more complicated due to the existence of coupled processes (internalization and recycling of these species). But if, for the sake of simplicity, these complications are ignored, it follows that, starting from 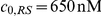 at 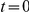 (see Table 2), there is a rapid loss of empty receptors during ligand input stimulation. This disappearance is parallel to the formation of ligand-receptor complexes, which causes a rapid increase in the 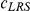-values. During the recovery phase, these species continue to be formed and to disappear, although at different rates because they are also modulated by internalization and recycling processes. At 50 s, the second ligand input perturbation starts, which again produces a sudden drop and increase in the curves of RS and LRS, respectively.

To conclude, the response of internalized ligand-receptor complexes to step inputs of ligand was computed both at the cell surface and in the center of the cell (curves a and b in Panel C). Both responses are similar but, as expected, the lag phase is longer in the center of the cell (curve b), revealing, on the one hand, that faster responses are attained at the cell surface (curve a), and on the other, that concentration gradients are also operating inside the cell. The LRI responses increase with time and reflect, through changes in their slopes and lag times, the outcome of the perturbations caused by ligand input. However, in this case, correlation with the profile shape of ligand input is very poor.

### Correlation between endocytic downregulation and ligand input

**Figure 16:**
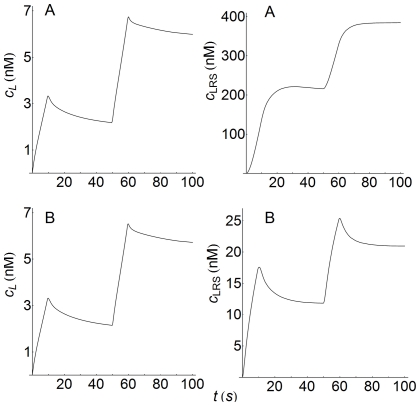
LRS response to step changes in ligand input rate with and without induced endocytosis. Concentration changes of L and LRS to step changes in the ligand input rate. Two inputs of 10 s duration separated by a recovery phase of 40 s were considered. In both inputs ligand enters the system at a constant rate of 1 nM/s. 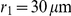, 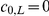. The ligand response was computed at the interface between cell surface and extracellular medium (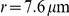). The surface ligand-receptor complex response was determined at 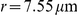. Panels A: 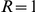. Panels B: 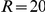. Other conditions as in Fig.3.

Finally, the correlation between endocytic downregulation and ligand input was examined. The results obtained at 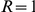 (absence of induced endocytosis) and 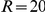 (enhanced induced endocytosis) for the responses of ligand and surface ligand-receptor complexes are displayed in Fig.16. When perturbations induced by ligand input were computed at the cell surface, the profiles obtained showed little dependence on the 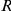-values (panels A and B in Fig.16). As regards the LRS response, for 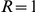 the system output showed little correlation with ligand input both in shape and magnitude (Panels A). However, for 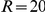 this correlation improved significantly in terms of shape and magnitude so that endocytic downregulation resulted in system outputs that follow variations in ligand inputs much more closely (panels B). This outcome seems general so that correlations improve as 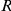 increases. These results agree with those obtained using a non-spatial model and can be attributed to the better information processing capacity of the system under these conditions [5]. However, from another point of view, the increasing internalization capacity of surface complexes (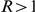) means there is greater perturbation of the system. Under these conditions, equilibrium in the surface-complex formation process is more difficult to attain. This results in faster LRS responses to ligand input perturbations which, in turn, improves the power for decoding input information. Conversely, under conditions that favor near equilibrium, the behavior of the system output is different. It is interesting to note in this respect that LRS responses for 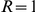 correlate better with ligand input in the outer regions of the extracellular medium where perturbation induced by ligand input has little influence (compare the 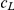-profile at 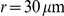 in Fig.15 with the LRS response in Fig.16, Panel A). Considering that information enters the cell as the result of ligand-receptor interactions at the cell surface, all these observations suggest that the correlation between ligand input and system response is greater in conditions that produce larger perturbations of the equilibrium 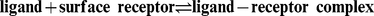.

### Biological Significance

Mechanisms of ligand-receptor induced endocytosis and their role in cell signaling has been the subject of a great number of recent publications in many relevant biological systems. However, the large amount of data available means it is impossible to reconcile them all whitin a single reasonable model. One of the major reasons for such discrepancies is the use of different methodologies by different laboratories. This underscores the importance of standardizing the methodological approaches for monitoring these processes, especially those that permit reliable quantification of the kinetics and diffusion rates. In fact, several authors have described the cell-density dependence of trafficking and signaling in different biological systems, but no general explanation has been proposed beyond contact inhibition phenomena [27]–[32]. Therefore, the results described here will contribute to explaining these discrepancies and to standardizing the experimental conditions necessary to obtain reliable quantitative results in this important field of the biology of cell signaling. The results presented in this paper suggest: 1) the advisability of going beyond simplified models that only study these processes by considering that the system is homogeneous; and 2) that experimental designs focused on obtaining data involved in the dynamics of signal perturbation propagation through the different domains as a function of space and time are very helpful for gaining insight into these complex systems.
